# Supplementary material for: RNA-Seq Analysis Reveals Localization-Associated Alternative Splicing across 13 Cell Lines
Source: Genes (Basel). 2020 Jul 18;11(7):820. doi: 10.3390/genes11070820 (PMC7397181; doi:10.3390/genes11070820)
Supplement: Supplementary file 1 [file genes-11-00820-s001.pdf]

Supplementary Data 1. RNA-seq data used in this study

Cell line	Tissue	Cytosolic_rep1 (C1)	Cytosolic_rep2 (C2)	Nuclear_rep1 (N1)	Nuclear_rep2 (N2)
A549	Lung	ENCFF000EKW	ENCFF000ELC	ENCFF000EMA	ENCFF000ELQ
		ENCFF000EKX	ENCFF000ELF	ENCFF000EMD	ENCFF000EMB
GM12878	Blood	ENCFF000EZH	ENCFF000EZF	ENCFF000FCG	ENCFF000FCH
		ENCFF000EZR	ENCFF000EZT	ENCFF000FCI	ENCFF000FCU
HeLa-S3	Cervix	ENCFF000FQK	ENCFF000FQW	ENCFF000FSU	ENCFF000FTB
		ENCFF000FQX	ENCFF000FQV	ENCFF000FTA	ENCFF000FTJ
HepG2	Liver	ENCFF000FXU	ENCFF000FYG	ENCFF000GAF	ENCFF000GAH
		ENCFF000FYF	ENCFF000FYH	ENCFF000GAT	ENCFF000GAS
HT1080	Connective tissue	ENCFF447UEE	ENCFF331FNA	ENCFF028JQU	ENCFF365ZYO
		ENCFF058MHC	ENCFF503XDE	ENCFF381YLG	ENCFF087HWA
HUVEC	Umbilical vein	ENCFF000GTX	ENCFF000GUJ	ENCFF000GWF	ENCFF000GWS
		ENCFF000GUH	ENCFF000GUI	ENCFF000GWT	ENCFF000GWV
IMR-90	Lung	ENCFF000HCB	ENCFF000HCI	ENCFF000HDD	ENCFF000HDE
		ENCFF000HCC	ENCFF000HCJ	ENCFF000HDF	ENCFF000HDH
MCF-7	Breast	ENCFF000HSB	ENCFF000HSC	ENCFF000HTE	ENCFF000HTD
		ENCFF000HSI	ENCFF000HSK	ENCFF000HVN	ENCFF000HVM
NHEK	Skin	ENCFF000IDC	ENCFF000IDF	ENCFF000IFF	ENCFF000IFI
		ENCFF000IDI	ENCFF000IDH	ENCFF000IFH	ENCFF000IFW
SK-MEL-5	Skin	ENCFF470ZTS	ENCFF687XKK	ENCFF005NLJ	ENCFF394ODW
		ENCFF299CXA	ENCFF570YTU	ENCFF635CQM	ENCFF365MLV
SK-N-DZ	Brain	ENCFF960DLP	ENCFF138HQU	ENCFF482SFO	ENCFF993CAL
		ENCFF288VWD	ENCFF822LCB	ENCFF691TRA	ENCFF689LXP
SK-N-SH	Brain	ENCFF000INM	ENCFF000INN	ENCFF000IOD	ENCFF000IOP
		ENCFF000INT	ENCFF000INV	ENCFF000IOQ	ENCFF000IPB
K562	Bone marrow	ENCFF000HJP	ENCFF000HJF	ENCFF000HOC	ENCFF000HOD
		ENCFF000HJW	ENCFF000HJX	ENCFF000HOE	ENCFF000HOQ

• The libraries were constructed from rRNA-depleted Ploy-A+ RNA (> 200bp in size)
• Strand-specific, PE101nt or PE76nt

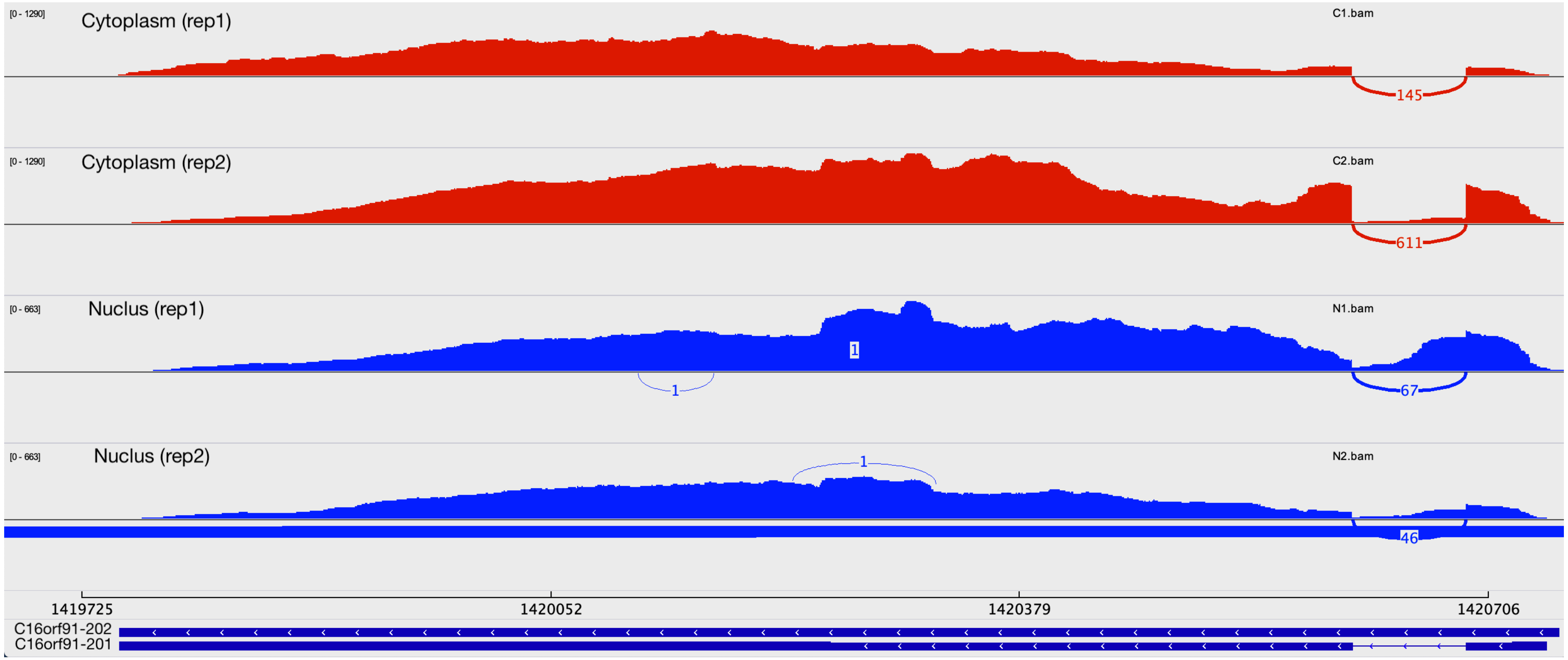

Supplementary Data 2. Read coverage of gene *C16orf91* in HeLa-S3. IGV (Robinson et al 2011) was used to visualize the read coverage.

Reference:

Robinson, J.T., Thorvaldsdóttir, H., Winckler, W., Guttman, M., Lander, E.S., Getz, G. and Mesirov, J.P., 2011. Integrative genomics viewer. Nature biotechnology, 29(1), pp.24-26.

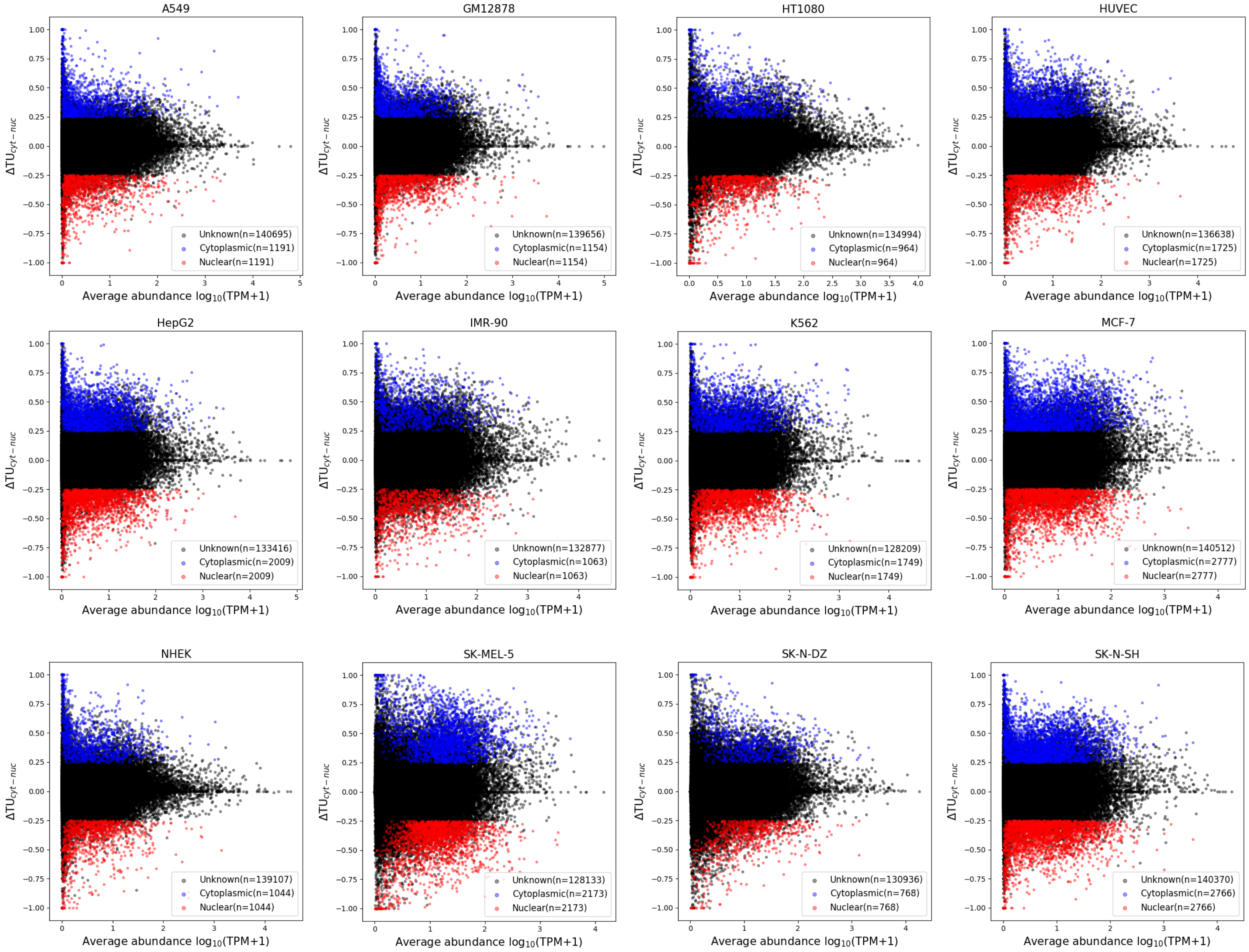

Supplementary Data 3. Transcriptome-wide identification of transcript variant switches in 12 human cell lines. We applied $|\Delta TU|>0.25$ and $p<0.05$ to filter out cytoplasmic($|\Delta TU|>0$, blue) and nuclear ($|\Delta TU|<0$, red) transcript variants. Unknown (black) are transcripts filtered as no significant.

Supplementary Data 4. List of switching genes identified across 13 cell lines in this study.

Online resource: https://doi.org/10.6084/m9.figshare.11999658

Supplementary Data 5. Gene Ontology enrichment analysis for switch genes (sheared, continued)

source	term_name	term_id	adjusted_p_value	negative_log10_of_adjusted_p_value
GO:MF	transmembrane signaling receptor activity	GO:0004888	1.55E-16	
GO:MF	molecular transducer activity	GO:0060089	5.59E-16	
GO:MF	signaling receptor activity	GO:0038023	5.68E-16	
GO:MF	G protein-coupled receptor activity	GO:0004930	6.56E-12	
GO:MF	passive transmembrane transporter activity	GO:0022803	0.000493696	
GO:MF	channel activity	GO:0015267	0.000493696	
GO:BP	multicellular organismal process	GO:0032501	3.31E-12	
GO:BP	G protein-coupled receptor signaling pathway	GO:0007186	2.10E-11	
GO:BP	system process	GO:0003008	3.32E-09	
GO:BP	sensory perception of chemical stimulus	GO:0007606	1.85E-07	
GO:BP	nervous system process	GO:0050877	2.73E-07	
GO:BP	detection of stimulus involved in sensory perception	GO:0050906	1.36162E-06	
GO:BP	detection of chemical stimulus involved in sensory perception	GO:0050907	1.61549E-06	
GO:BP	signaling	GO:0023052	2.52304E-06	
GO:BP	signal transduction	GO:0007165	1.66204E-05	
GO:BP	detection of chemical stimulus	GO:0009593	2.20905E-05	
GO:BP	sensory perception	GO:0007600	2.65966E-05	
GO:BP	cell communication	GO:0007154	2.81869E-05	
GO:BP	defense response	GO:0006952	6.05683E-05	
GO:BP	detection of stimulus	GO:0051606	6.68613E-05	
GO:BP	response to external stimulus	GO:0009605	0.000100343	
GO:BP	multicellular organism development	GO:0007275	0.000100343	
GO:BP	adaptive immune response	GO:0002250	0.000171355	
GO:BP	cell differentiation	GO:0030154	0.000179869	
GO:BP	system development	GO:0048731	0.000207557	
GO:BP	ion transport	GO:0006811	0.000405413	
GO:BP	positive regulation of immune system process	GO:0002684	0.000439648	
GO:BP	anatomical structure development	GO:0048856	0.000518076	
GO:BP	regulation of multicellular organismal process	GO:0051239	0.000537973	
GO:BP	defense response to bacterium	GO:0042742	0.000820237	
GO:CC	plasma membrane	GO:0005886	3.50E-13	
GO:CC	cell periphery	GO:0071944	4.22E-13	
GO:CC	extracellular region	GO:0005576	1.17977E-05	
GO:CC	plasma membrane region	GO:0098590	0.000637715	
GO:CC	intrinsic component of membrane	GO:0031224	0.000637715	

GO(Gene Ontology) enrichment analysis for 1219 sheared switching genes (detected as switch gene ≥ 5 cell lines in this study) was conducted by g:Profiler(PMID: 31066453) with following parameters:

- Hypergeometric test
- Benjamini–Hochberg correction
- FDR(False Discovery Rate) < 0.001

* MF, BP, CC represent Molecular Function, Biological Process, Cellular Component, respectively.

Supplementary Data 5. Gene Ontology enrichment analysis for switch genes (A549, continued)

source	term_name	term_id	adjusted_p_value	negative_log10_of_adjusted_p_value
GO:MF	transmembrane signaling receptor activity	GO:0004888	2.58E-09	
GO:MF	signaling receptor activity	GO:0038023	9.26E-08	
GO:MF	molecular transducer activity	GO:0060089	9.26E-08	
GO:MF	G protein-coupled receptor activity	GO:0004930	1.27132E-05	
GO:BP	G protein-coupled receptor signaling pathway	GO:0007186	1.535E-05	
GO:BP	sensory perception of chemical stimulus	GO:0007606	2.3181E-05	
GO:BP	detection of chemical stimulus involved in sensory perception	GO:0050907	2.3181E-05	
GO:BP	sensory perception of smell	GO:0007608	3.06052E-05	
GO:BP	detection of chemical stimulus	GO:0009593	3.06052E-05	
GO:BP	detection of stimulus involved in sensory perception	GO:0050906	8.11614E-05	
GO:CC	no significant results			

GO(Gene Ontology) enrichment analysis for 1191 switching genes in A549 was conducted by g:Profiler(PMID: 31066453) with following parameters:

- Hypergeometric test
- Benjamini–Hochberg correction
- FDR(False Discovery Rate) < 0.001

* MF, BP, CC represent Molecular Function, Biological Process, Cellular Component, respectively.

Supplementary Data 5. Gene Ontology enrichment analysis for switch genes (GM12878, continued)

source	term_name	term_id	adjusted_p_value	negative_log10_of_adjusted_p_value
GO:MF	G protein-coupled receptor activity	GO:0004930	4.27792E-05	
GO:BP	G protein-coupled receptor signaling pathway	GO:0007186	3.80158E-05	
GO:BP	system process	GO:0003008	0.000132801	
GO:CC	no significant results			

GO(Gene Ontology) enrichment analysis for 1154 switching genes in GM12878 was conducted by g:Profiler(PMID: 31066453) with following parameters:

- Hypergeometric test
- Benjamini–Hochberg correction
- FDR(False Discovery Rate) < 0.001

Supplementary Data 5. Gene Ontology enrichment analysis for switch genes (HT1080, continued)

source	term_name	term_id	adjusted_p_value	negative_log10_of_adjusted_p_value
GO:MF	transmembrane signaling receptor activity	GO:0004888	6.49781E-05	
GO:MF	G protein-coupled receptor activity	GO:0004930	0.000178858	
GO:MF	signaling receptor activity	GO:0038023	0.000211021	
GO:MF	molecular transducer activity	GO:0060089	0.000211021	
GO:BP	signal transduction	GO:0007165	4.50381E-06	
GO:BP	multicellular organismal process	GO:0032501	4.50381E-06	
GO:BP	G protein-coupled receptor signaling pathway	GO:0007186	5.06491E-06	
GO:BP	signaling	GO:0023052	5.06491E-06	
GO:BP	cell communication	GO:0007154	2.71825E-05	
GO:CC	extracellular region	GO:0005576	4.51E-12	
GO:CC	extracellular space	GO:0005615	5.10E-10	
GO:CC	cell periphery	GO:0071944	1.51052E-06	
GO:CC	plasma membrane	GO:0005886	2.08264E-06	
GO:CC	extracellular organelle	GO:0043230	6.65978E-05	
GO:CC	extracellular vesicle	GO:1903561	6.65978E-05	
GO:CC	extracellular exosome	GO:0070062	9.1387E-05	

GO(Gene Ontology) enrichment analysis for 964 switching genes in HT1080 was conducted by g:Profiler(PMID: 31066453) with following parameters:

- Hypergeometric test
- Benjamini–Hochberg correction
- FDR(False Discovery Rate) < 0.001

Supplementary Data 5. Gene Ontology enrichment analysis for switch genes (HUVEC, continued)

source	term_name	term_id	adjusted_p_value	negative_log10_of_adjusted_p_value
GO:MF	transmembrane signaling receptor activity	GO:0004888	1.04E-08	
GO:MF	G protein-coupled receptor activity	GO:0004930	1.04E-08	
GO:MF	signaling receptor activity	GO:0038023	1.73E-07	
GO:MF	molecular transducer activity	GO:0060089	1.96E-07	
GO:BP	detection of stimulus involved in sensory perception	GO:0050906	5.37E-10	
GO:BP	detection of chemical stimulus involved in sensory perception	GO:0050907	9.09E-10	
GO:BP	detection of stimulus	GO:0051606	5.62E-09	
GO:BP	sensory perception of chemical stimulus	GO:0007606	2.26E-08	
GO:BP	sensory perception of smell	GO:0007608	2.26E-08	
GO:BP	multicellular organismal process	GO:0032501	1.40E-07	
GO:BP	system process	GO:0003008	2.72E-07	
GO:BP	detection of chemical stimulus	GO:0009593	2.90E-07	
GO:BP	sensory perception	GO:0007600	4.30E-07	
GO:BP	nervous system process	GO:0050877	1.37468E-05	
GO:BP	G protein-coupled receptor signaling pathway	GO:0007186	7.79881E-05	
GO:BP	cell differentiation	GO:0030154	0.000238532	
GO:CC	extracellular region	GO:0005576	2.7509E-06	
GO:CC	extracellular space	GO:0005615	0.000209584	

GO(Gene Ontology) enrichment analysis for 1725 switching genes in HUVEC was conducted by g:Profiler(PMID: 31066453) with following parameters:

- Hypergeometric test
- Benjamini–Hochberg correction
- FDR(False Discovery Rate) < 0.001

* MF, BP, CC represent Molecular Function, Biological Process, Cellular Component, respectively.

Supplementary Data 5. Gene Ontology enrichment analysis for switch genes (HeLa-S3, continued)

source	term_name	term_id	adjusted_p_value	-log10 (adjusted_p_value)
GO:MF	transmembrane signaling receptor activity	GO:0004888	6.36E-20	
GO:MF	signaling receptor activity	GO:0038023	3.32E-18	
GO:MF	molecular transducer activity	GO:0060089	4.04E-18	
GO:MF	G protein-coupled receptor activity	GO:0004930	1.43E-16	
GO:MF	gated channel activity	GO:0022836	0.00014682	
GO:BP	G protein-coupled receptor signaling pathway	GO:0007186	4.22E-14	
GO:BP	detection of chemical stimulus involved in sensory perception	GO:0050907	4.22E-13	
GO:BP	detection of stimulus involved in sensory perception	GO:0050906	5.95E-13	
GO:BP	sensory perception of chemical stimulus	GO:0007606	6.90E-13	
GO:BP	multicellular organismal process	GO:0032501	7.57E-13	
GO:BP	nervous system process	GO:0050877	1.73E-12	
GO:BP	sensory perception	GO:0007600	4.13E-12	
GO:BP	sensory perception of smell	GO:0007608	1.13E-11	
GO:BP	detection of chemical stimulus	GO:0009593	1.59E-11	
GO:BP	system process	GO:0003008	4.10E-11	
GO:BP	detection of stimulus	GO:0051606	2.85E-07	
GO:BP	adaptive immune response	GO:0002250	4.41341E-05	
GO:BP	signaling	GO:0023052	0.000978609	
GO:CC	plasma membrane	GO:0005886	3.22E-20	
GO:CC	cell periphery	GO:0071944	6.49E-20	
GO:CC	integral component of plasma membrane	GO:0005887	1.44E-09	
GO:CC	integral component of membrane	GO:0016021	1.84E-09	
GO:CC	intrinsic component of membrane	GO:0031224	3.40E-09	
GO:CC	intrinsic component of plasma membrane	GO:0031226	6.90E-09	
GO:CC	receptor complex	GO:0043235	3.00457E-06	
GO:CC	plasma membrane region	GO:0098590	2.56347E-05	
GO:CC	extracellular matrix	GO:0031012	3.55608E-05	
GO:CC	plasma membrane protein complex	GO:0098797	0.000121469	
GO:CC	plasma membrane signaling receptor complex	GO:0098802	0.000238214	
GO:CC	collagen-containing extracellular matrix	GO:0062023	0.000679446	
GO:CC	extracellular region	GO:0005576	0.000679446	
GO:CC	synaptic membrane	GO:0097060	0.000679446	

GO(Gene Ontology) enrichment analysis for 2073 switching genes in HeLa-S3 was conducted by g:Profiler(PMID: 31066453) with following parameters:

- Hypergeometric test
- Benjamini–Hochberg correction
- FDR(False Discovery Rate) < 0.001

* MF, BP, CC represent Molecular Function, Biological Process, Cellular Component, respectively.

Supplementary Data 5. Gene Ontology enrichment analysis for switch genes (HepG2, continued)

source	term_name	term_id	adjusted_p_value	negative_log10_of_adjusted_p_value
GO:MF	transmembrane signaling receptor activity	GO:0004888	3.54E-15	
GO:MF	G protein-coupled receptor activity	GO:0004930	9.45E-13	
GO:MF	olfactory receptor activity	GO:0004984	1.15E-12	
GO:MF	signaling receptor activity	GO:0038023	1.15E-12	
GO:MF	molecular transducer activity	GO:0060089	1.15E-12	
GO:BP	G protein-coupled receptor signaling pathway	GO:0007186	5.33E-14	
GO:BP	sensory perception of chemical stimulus	GO:0007606	9.52E-14	
GO:BP	sensory perception of smell	GO:0007608	1.60E-12	
GO:BP	detection of chemical stimulus involved in sensory perception	GO:0050907	5.18E-12	
GO:BP	detection of chemical stimulus involved in sensory perception of smell	GO:0050911	1.43E-11	
GO:BP	detection of chemical stimulus	GO:0009593	2.53E-10	
GO:BP	system process	GO:0003008	4.56E-09	
GO:BP	multicellular organismal process	GO:0032501	7.88E-09	
GO:BP	detection of stimulus involved in sensory perception	GO:0050906	9.69E-09	
GO:BP	sensory perception	GO:0007600	1.23E-08	
GO:BP	nervous system process	GO:0050877	2.19E-08	
GO:BP	detection of stimulus	GO:0051606	8.61E-08	
GO:BP	signaling	GO:0023052	6.64E-07	
GO:BP	signal transduction	GO:0007165	9.84594E-06	
GO:BP	cell communication	GO:0007154	1.09238E-05	
GO:BP	keratinization	GO:0031424	0.000190632	
GO:BP	regulation of response to stimulus	GO:0048583	0.000408547	
GO:BP	humoral immune response	GO:0006959	0.000504249	
GO:BP	keratinocyte differentiation	GO:0030216	0.000527629	
GO:BP	gene silencing by miRNA	GO:0035195	0.000527629	
GO:CC	cell periphery	GO:0071944	4.18E-14	
GO:CC	plasma membrane	GO:0005886	1.57E-13	
GO:CC	integral component of membrane	GO:0016021	1.44303E-06	
GO:CC	intrinsic component of membrane	GO:0031224	2.02693E-06	
GO:CC	integral component of plasma membrane	GO:0005887	0.000194967	
GO:CC	intrinsic component of plasma membrane	GO:0031226	0.00034656	
GO:CC	plasma membrane protein complex	GO:0098797	0.000698663	

GO(Gene Ontology) enrichment analysis for 2009 switching genes in HepG2 was conducted by g:Profiler(PMID: 31066453) with following parameters:

- Hypergeometric test
- Benjamini–Hochberg correction
- FDR(False Discovery Rate) < 0.001

* MF, BP, CC represent Molecular Function, Biological Process, Cellular Component, respectively.

Supplementary Data 5. Gene Ontology enrichment analysis for switch genes (IMR-90, continued)

source	term_name	term_id	adjusted_p_value	negative_log10_of_adjusted_p_value
GO:MF	transmembrane signaling receptor activity	GO:0004888	5.05E-11	
GO:MF	signaling receptor activity	GO:0038023	7.05E-11	
GO:MF	molecular transducer activity	GO:0060089	1.38E-10	
GO:MF	G protein-coupled receptor activity	GO:0004930	3.27E-09	
GO:MF	olfactory receptor activity	GO:0004984	5.7643E-05	
GO:BP	multicellular organismal process	GO:0032501	3.2905E-06	
GO:BP	sensory perception	GO:0007600	4.58447E-05	
GO:BP	system process	GO:0003008	6.72981E-05	
GO:BP	nervous system process	GO:0050877	0.000186145	
GO:BP	G protein-coupled receptor signaling pathway	GO:0007186	0.000239019	
GO:BP	detection of stimulus involved in sensory perception	GO:0050906	0.000612089	
GO:BP	detection of chemical stimulus involved in sensory perception of smell	GO:0050911	0.000612089	
GO:BP	sensory perception of chemical stimulus	GO:0007606	0.000618172	
GO:BP	detection of chemical stimulus involved in sensory perception	GO:0050907	0.000618172	
GO:CC	plasma membrane	GO:0005886	1.24E-07	
GO:CC	cell periphery	GO:0071944	1.24E-07	

GO(Gene Ontology) enrichment analysis for 1063 switching genes in IMR-90 was conducted by g:Profiler(PMID: 31066453) with following parameters:

- Hypergeometric test
- Benjamini–Hochberg correction
- FDR(False Discovery Rate) < 0.001

* MF, BP, CC represent Molecular Function, Biological Process, Cellular Component, respectively.

Supplementary Data 5. Gene Ontology enrichment analysis for switch genes (K562, continued)

source	term_name	term_id	adjusted_p_value	negative_log10_of_adjusted_p_value
GO:MF	G protein-coupled receptor activity	GO:0004930	6.83E-11	
GO:MF	transmembrane signaling receptor activity	GO:0004888	3.65E-09	
GO:MF	signaling receptor activity	GO:0038023	2.52E-08	
GO:MF	molecular transducer activity	GO:0060089	2.89E-08	
GO:BP	G protein-coupled receptor signaling pathway	GO:0007186	1.94E-15	
GO:BP	system process	GO:0003008	4.80E-12	
GO:BP	nervous system process	GO:0050877	1.01E-10	
GO:BP	detection of chemical stimulus	GO:0009593	1.18E-09	
GO:BP	detection of chemical stimulus involved in sensory perception	GO:0050907	1.18E-09	
GO:BP	multicellular organismal process	GO:0032501	9.58E-09	
GO:BP	detection of stimulus involved in sensory perception	GO:0050906	1.36E-08	
GO:BP	sensory perception of smell	GO:0007608	3.31E-08	
GO:BP	sensory perception	GO:0007600	2.87E-07	
GO:BP	sensory perception of chemical stimulus	GO:0007606	5.46E-07	
GO:BP	detection of stimulus	GO:0051606	1.31782E-06	
GO:BP	adaptive immune response	GO:0002250	0.000259623	
GO:CC	cell periphery	GO:0071944	3.90E-09	
GO:CC	plasma membrane	GO:0005886	4.12E-09	
GO:CC	extracellular region	GO:0005576	0.000163539	
GO:CC	intrinsic component of membrane	GO:0031224	0.000205003	
GO:CC	extracellular matrix	GO:0031012	0.000216974	
GO:CC	integral component of membrane	GO:0016021	0.000295277	

GO(Gene Ontology) enrichment analysis for 1749 switching genes in K562 was conducted by g:Profiler(PMID: 31066453) with following parameters:

- Hypergeometric test
- Benjamini–Hochberg correction
- FDR(False Discovery Rate) < 0.001

* MF, BP, CC represent Molecular Function, Biological Process, Cellular Component, respectively.

Supplementary Data 5. Gene Ontology enrichment analysis for switch genes (MCF-7, continued)

source	term_name	term_id	adjusted_p_value	negative_log10_of_adjusted_p_value
GO:MF	transmembrane signaling receptor activity	GO:0004888	3.29E-22	
GO:MF	signaling receptor activity	GO:0038023	9.46E-20	
GO:MF	molecular transducer activity	GO:0060089	9.46E-20	
GO:MF	G protein-coupled receptor activity	GO:0004930	1.26E-17	
GO:MF	receptor regulator activity	GO:0030545	0.000673771	
GO:BP	detection of chemical stimulus involved in sensory perception	GO:0050907	9.20E-19	
GO:BP	detection of stimulus involved in sensory perception	GO:0050906	4.21E-17	
GO:BP	detection of chemical stimulus	GO:0009593	4.21E-17	
GO:BP	multicellular organismal process	GO:0032501	1.97E-15	
GO:BP	G protein-coupled receptor signaling pathway	GO:0007186	8.82E-15	
GO:BP	sensory perception of chemical stimulus	GO:0007606	3.28E-14	
GO:BP	sensory perception of smell	GO:0007608	1.02E-12	
GO:BP	detection of stimulus	GO:0051606	5.08E-12	
GO:BP	sensory perception	GO:0007600	5.29E-12	
GO:BP	system process	GO:0003008	1.22E-11	
GO:BP	nervous system process	GO:0050877	2.33E-11	
GO:BP	gene silencing by miRNA	GO:0035195	2.77583E-05	
GO:BP	posttranscriptional gene silencing	GO:0016441	3.61883E-05	
GO:BP	posttranscriptional gene silencing by RNA	GO:0035194	3.66099E-05	
GO:BP	response to external stimulus	GO:0009605	7.15327E-05	
GO:BP	signaling	GO:0023052	7.15327E-05	
GO:BP	adaptive immune response	GO:0002250	9.03152E-05	
GO:BP	regulation of multicellular organismal process	GO:0051239	0.000169194	
GO:BP	cell communication	GO:0007154	0.000223032	
GO:BP	humoral immune response	GO:0006959	0.000229014	
GO:BP	gene silencing by RNA	GO:0031047	0.000233736	
GO:BP	keratinocyte differentiation	GO:0030216	0.000373816	
GO:BP	keratinization	GO:0031424	0.000471737	
GO:BP	system development	GO:0048731	0.000493893	
GO:BP	regulation of developmental process	GO:0050793	0.000493893	
GO:BP	defense response	GO:0006952	0.000572092	
GO:BP	multicellular organism development	GO:0007275	0.000586089	
GO:BP	signal transduction	GO:0007165	0.000639678	
GO:BP	positive regulation of developmental process	GO:0051094	0.000859708	
GO:BP	defense response to bacterium	GO:0042742	0.000886349	
GO:BP	G protein-coupled receptor signaling pathway, coupled to cyclic nucleotide second messenger	GO:0007187	0.000886349	
GO:BP	anatomical structure development	GO:0048856	0.000946523	
GO:CC	plasma membrane	GO:0005886	2.37E-12	
GO:CC	cell periphery	GO:0071944	2.37E-12	
GO:CC	receptor complex	GO:0043235	1.57E-07	
GO:CC	intrinsic component of membrane	GO:0031224	1.81786E-05	
GO:CC	integral component of plasma membrane	GO:0005887	2.17165E-05	
GO:CC	extracellular region	GO:0005576	2.17165E-05	
GO:CC	integral component of membrane	GO:0016021	2.30812E-05	
GO:CC	intrinsic component of plasma membrane	GO:0031226	2.30812E-05	
GO:CC	intermediate filament	GO:0005882	6.0376E-05	
GO:CC	plasma membrane protein complex	GO:0098797	0.000166003	
GO:CC	plasma membrane region	GO:0098590	0.000387874	

GO(Gene Ontology) enrichment analysis for 2777 switching genes in MCF-7 was conducted by g:Profiler(PMID: 31066453) with following parameters:

- Hypergeometric test
- Benjamini-Hochberg correction
- FDR(False Discovery Rate) < 0.001

* MF, BP, CC represent Molecular Function, Biological Process, Cellular Component, respectively.

Supplementary Data 5. Gene Ontology enrichment analysis for switch genes (NHEK, continued)

source	term_name	term_id	adjusted_p_value	negative_log10_of_adjusted_p_value
GO:MF	transmembrane signaling receptor activity	GO:0004888	0.000223642	
GO:MF	signaling receptor activity	GO:0038023	0.000223642	
GO:MF	molecular transducer activity	GO:0060089	0.000223642	
GO:MF	olfactory receptor activity	GO:0004984	0.000669489	
GO:BP	multicellular organismal process	GO:0032501	0.000809884	
GO:CC	extracellular region	GO:0005576	0.000461703	
GO:CC	plasma membrane	GO:0005886	0.00049703	
GO:CC	cell periphery	GO:0071944	0.000666079	

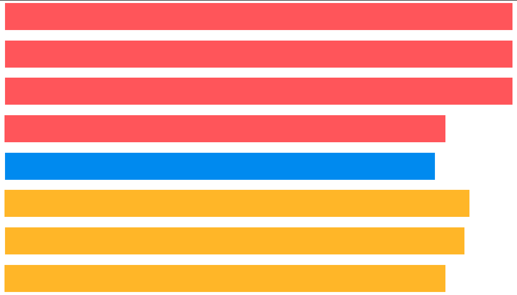

GO(Gene Ontology) enrichment analysis for 2777 switching genes in NHEK was conducted by g:Profiler(PMID: 31066453) with following parameters:

- Hypergeometric test
- Benjamini–Hochberg correction
- FDR(False Discovery Rate) < 0.001

Supplementary Data 5. Gene Ontology enrichment analysis for switch genes (SK-MEL-5, continued)

source	term_name	term_id	adjusted_p_value	negative_log10_of_adjusted_p_value
GO:MF	transmembrane signaling receptor activity	GO:0004888	2.50E-20	
GO:MF	G protein-coupled receptor activity	GO:0004930	2.50E-20	
GO:MF	signaling receptor activity	GO:0038023	2.50E-20	
GO:MF	molecular transducer activity	GO:0060089	2.50E-20	
GO:MF	receptor regulator activity	GO:0030545	4.34223E-05	
GO:MF	passive transmembrane transporter activity	GO:0022803	0.000322256	
GO:MF	channel activity	GO:0015267	0.000322256	
GO:MF	signaling receptor activator activity	GO:0030546	0.000481617	
GO:MF	receptor ligand activity	GO:0048018	0.000631058	
GO:MF	DNA-binding transcription factor activity	GO:0003700	0.000739906	
GO:BP	G protein-coupled receptor signaling pathway	GO:0007186	3.38E-17	
GO:BP	detection of stimulus involved in sensory perception	GO:0050906	3.39E-17	
GO:BP	detection of chemical stimulus involved in sensory perception	GO:0050907	3.73E-16	
GO:BP	multicellular organismal process	GO:0032501	1.60E-15	
GO:BP	system process	GO:0003008	1.60E-15	
GO:BP	detection of chemical stimulus	GO:0009593	2.10E-15	
GO:BP	sensory perception	GO:0007600	5.11E-15	
GO:BP	sensory perception of chemical stimulus	GO:0007606	4.57E-14	
GO:BP	nervous system process	GO:0050877	1.96E-13	
GO:BP	sensory perception of smell	GO:0007608	3.80E-12	
GO:BP	detection of stimulus	GO:0051606	4.66E-11	
GO:BP	adaptive immune response	GO:0002250	1.89E-08	
GO:BP	signaling	GO:0023052	1.21971E-06	
GO:BP	humoral immune response	GO:0006959	9.91038E-06	
GO:BP	defense response to bacterium	GO:0042742	1.30477E-05	
GO:BP	cell communication	GO:0007154	1.33747E-05	
GO:BP	signal transduction	GO:0007165	4.62854E-05	
GO:BP	response to bacterium	GO:0009617	8.22478E-05	
GO:BP	cell differentiation	GO:0030154	0.000132096	
GO:BP	cell adhesion	GO:0007155	0.000194183	
GO:BP	biological adhesion	GO:0022610	0.000230205	
GO:BP	cellular developmental process	GO:0048869	0.000259699	
GO:BP	keratinization	GO:0031424	0.000331074	
GO:BP	epidermal cell differentiation	GO:0009913	0.000362886	
GO:BP	G protein-coupled receptor signaling pathway, coupled to cyclic nucleotide second messenger	GO:0007187	0.000362886	
GO:BP	regulation of transmembrane transport	GO:0034762	0.000362886	
GO:BP	biological regulation	GO:0065007	0.000400538	
GO:BP	inflammatory response	GO:0006954	0.000400538	
GO:BP	humoral immune response mediated by circulating immunoglobulin	GO:0002455	0.000400538	
GO:BP	regulation of ion transport	GO:0043269	0.000451217	
GO:BP	keratinocyte differentiation	GO:0030216	0.000664627	
GO:BP	regulation of ion transmembrane transport	GO:0034765	0.000944702	
GO:BP	regulation of cellular process	GO:0050794	0.000998044	
GO:BP	response to stimulus	GO:0050896	0.000998044	
GO:CC	plasma membrane	GO:0005886	2.21E-24	
GO:CC	cell periphery	GO:0071944	3.52E-24	
GO:CC	intrinsic component of plasma membrane	GO:0031226	6.58E-09	
GO:CC	integral component of plasma membrane	GO:0005887	3.39E-08	
GO:CC	intrinsic component of membrane	GO:0031224	1.36001E-06	
GO:CC	plasma membrane protein complex	GO:0098797	1.73042E-06	
GO:CC	integral component of membrane	GO:0016021	8.63394E-06	
GO:CC	extracellular region	GO:0005576	0.00012106	
GO:CC	receptor complex	GO:0043235	0.000138826	
GO:CC	intermediate filament	GO:0005882	0.000227515	
GO:CC	plasma membrane region	GO:0098590	0.000400338	

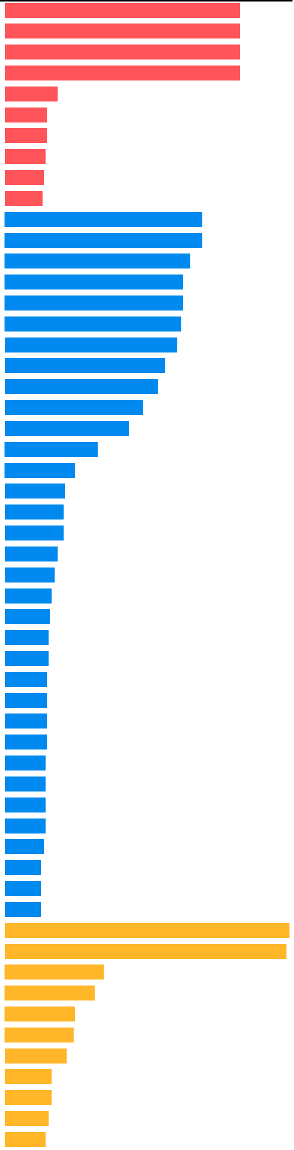

GO(Gene Ontology) enrichment analysis for 2173 switching genes in SK-MEL-5 was conducted by g:Profiler(PMID: 31066453) with following parameters:

- Hypergeometric test
- Benjamini–Hochberg correction
- FDR(False Discovery Rate) < 0.001

* MF, BP, CC represent Molecular Function, Biological Process, Cellular Component, respectively.

Supplementary Data 5. Gene Ontology enrichment analysis for switch genes (SK-N-DZ, continued)

source	term_name	term_id	adjusted_p_value	negative_log10_of_adjusted_p_value
GO:MF	transmembrane signaling receptor activity	GO:0004888	0.000446565	
GO:BP	multicellular organismal process	GO:0032501	2.27025E-05	
GO:CC	no significant results			

GO(Gene Ontology) enrichment analysis for 768 switching genes in SK-N-DZ was conducted by g:Profiler(PMID: 31066453) with following parameters:

- Hypergeometric test
- Benjamini–Hochberg correction
- FDR(False Discovery Rate) < 0.001

Supplementary Data 5. Gene Ontology enrichment analysis for switch genes (SK-N-SH)

source	term_name	term_id	adjusted_p_value	negative_log10_of_adjusted_p_value
GO:MF	transmembrane signaling receptor activity	GO:0004888	3.19E-16	
GO:MF	signaling receptor activity	GO:0038023	3.19E-16	
GO:MF	G protein-coupled receptor activity	GO:0004930	5.19E-16	
GO:MF	molecular transducer activity	GO:0060089	5.19E-16	
GO:MF	olfactory receptor activity	GO:0004984	2.89E-15	
GO:MF	antigen binding	GO:0003823	0.000131859	
GO:BP	detection of chemical stimulus	GO:0009593	3.47E-15	
GO:BP	detection of chemical stimulus involved in sensory perception	GO:0050907	8.52E-15	
GO:BP	G protein-coupled receptor signaling pathway	GO:0007186	8.77E-15	
GO:BP	sensory perception of chemical stimulus	GO:0007606	1.30E-14	
GO:BP	detection of chemical stimulus involved in sensory perception of smell	GO:0050911	6.08E-14	
GO:BP	sensory perception of smell	GO:0007608	2.32E-12	
GO:BP	nervous system process	GO:0050877	5.53E-12	
GO:BP	detection of stimulus involved in sensory perception	GO:0050906	8.87E-12	
GO:BP	system process	GO:0003008	1.37E-10	
GO:BP	sensory perception	GO:0007600	2.82E-10	
GO:BP	multicellular organismal process	GO:0032501	1.60E-09	
GO:BP	detection of stimulus	GO:0051606	2.96E-07	
GO:BP	keratinization	GO:0031424	5.33503E-06	
GO:BP	defense response to bacterium	GO:0042742	2.54298E-05	
GO:BP	adaptive immune response	GO:0002250	8.74689E-05	
GO:BP	signaling	GO:0023052	0.000829655	
GO:BP	humoral immune response mediated by circulating immunoglobulin	GO:0002455	0.000881868	
GO:CC	plasma membrane	GO:0005886	2.24E-19	
GO:CC	cell periphery	GO:0071944	2.11E-18	
GO:CC	extracellular region	GO:0005576	1.40E-08	
GO:CC	intrinsic component of membrane	GO:0031224	6.73E-08	
GO:CC	integral component of membrane	GO:0016021	7.91E-08	
GO:CC	membrane	GO:0016020	2.26958E-05	
GO:CC	plasma membrane region	GO:0098590	8.71218E-05	
GO:CC	receptor complex	GO:0043235	8.71218E-05	
GO:CC	extracellular space	GO:0005615	8.71218E-05	
GO:CC	cell surface	GO:0009986	8.72698E-05	
GO:CC	intrinsic component of plasma membrane	GO:0031226	0.000264325	
GO:CC	integral component of plasma membrane	GO:0005887	0.000455522	

GO(Gene Ontology) enrichment analysis for 2766 switching genes in SK-N-SH was conducted by g:Profiler(PMID: 31066453) with following parameters:

- Hypergeometric test
- Benjamini-Hochberg correction
- FDR(False Discovery Rate) < 0.001

* MF, BP, CC represent Molecular Function, Biological Process, Cellular Component, respectively.

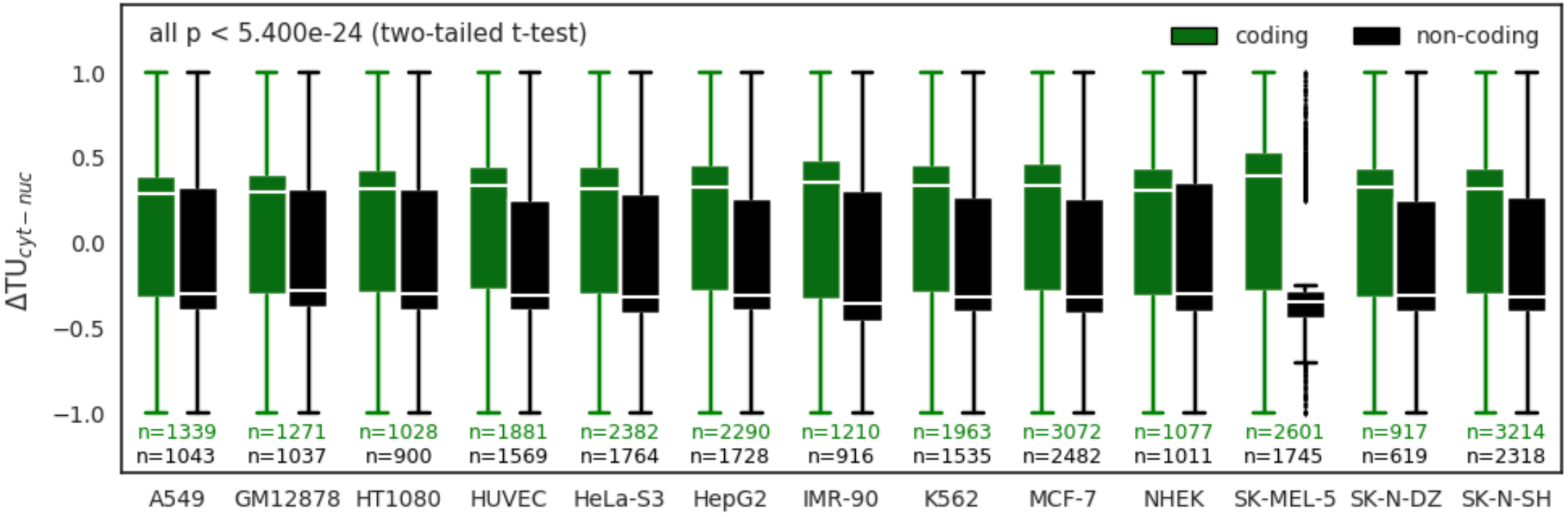

Supplementary Data 6. Comparison of ΔTU between protein-coding transcripts (green) and non-coding transcripts (black). Protein-coding transcripts possess higher ΔTU scores, which is consistent with our understanding that the transcript encoding protein is more prone to be located in the cytoplasm. Based on two-tailed t-test, we calculated the significant level of difference between ΔTU in protein-coding and non-coding transcripts in each cell line. The largest p value is 5.400e-24.

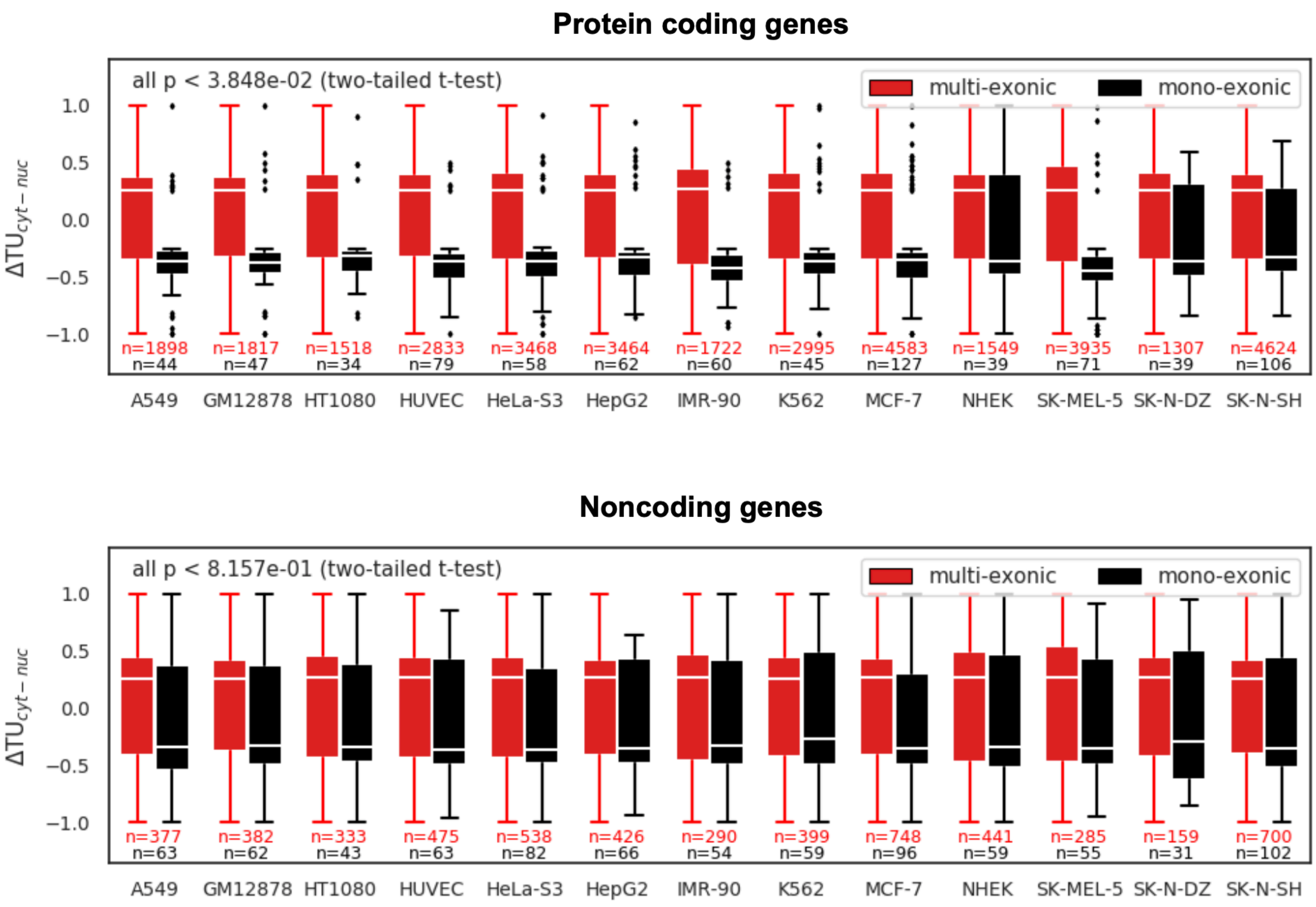

Supplementary Data 7. Comparison of ΔTU between mono-exonic (black) and multi-exonic (red) transcripts across 13 cell lines. Top and bottom panels for protein-coding and noncoding genes, respectively. ΔTU shows a significant positive correlation with splicing, indicating that splicing appears to be a dominant factor for RNA export from the nucleus. Based on two-tailed t-test, we calculated the significant level of difference between ΔTU in multi-exonic and mono-exonic transcripts in each cell line. The largest p value is 3.848e-02 and 8.157e-01 for protein-coding and non-coding genes, respectively.

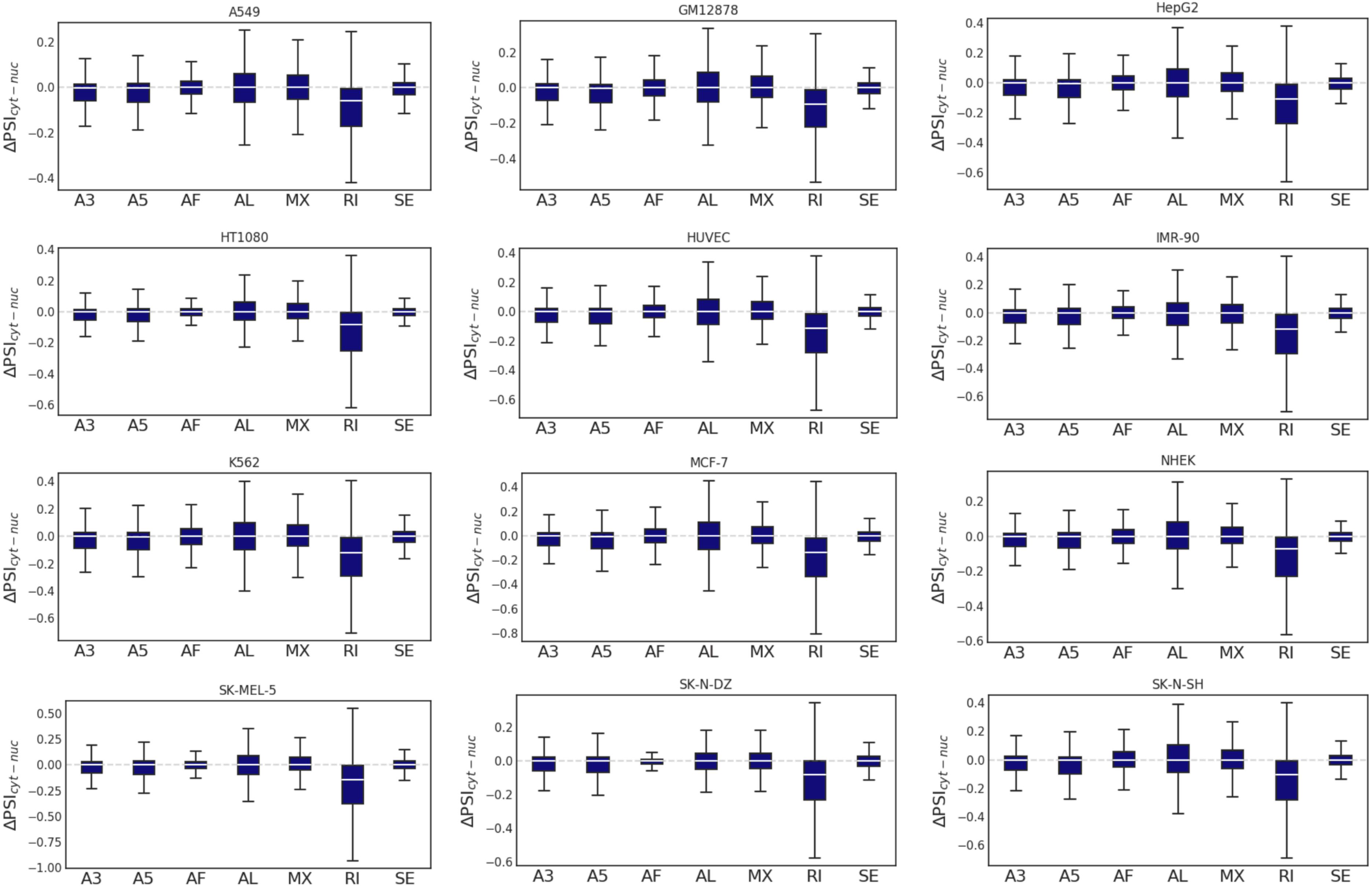

Supplementary Data 8. Comparison of alternative splicing patterns between cytoplasmic and nuclear transcripts. (all genes)

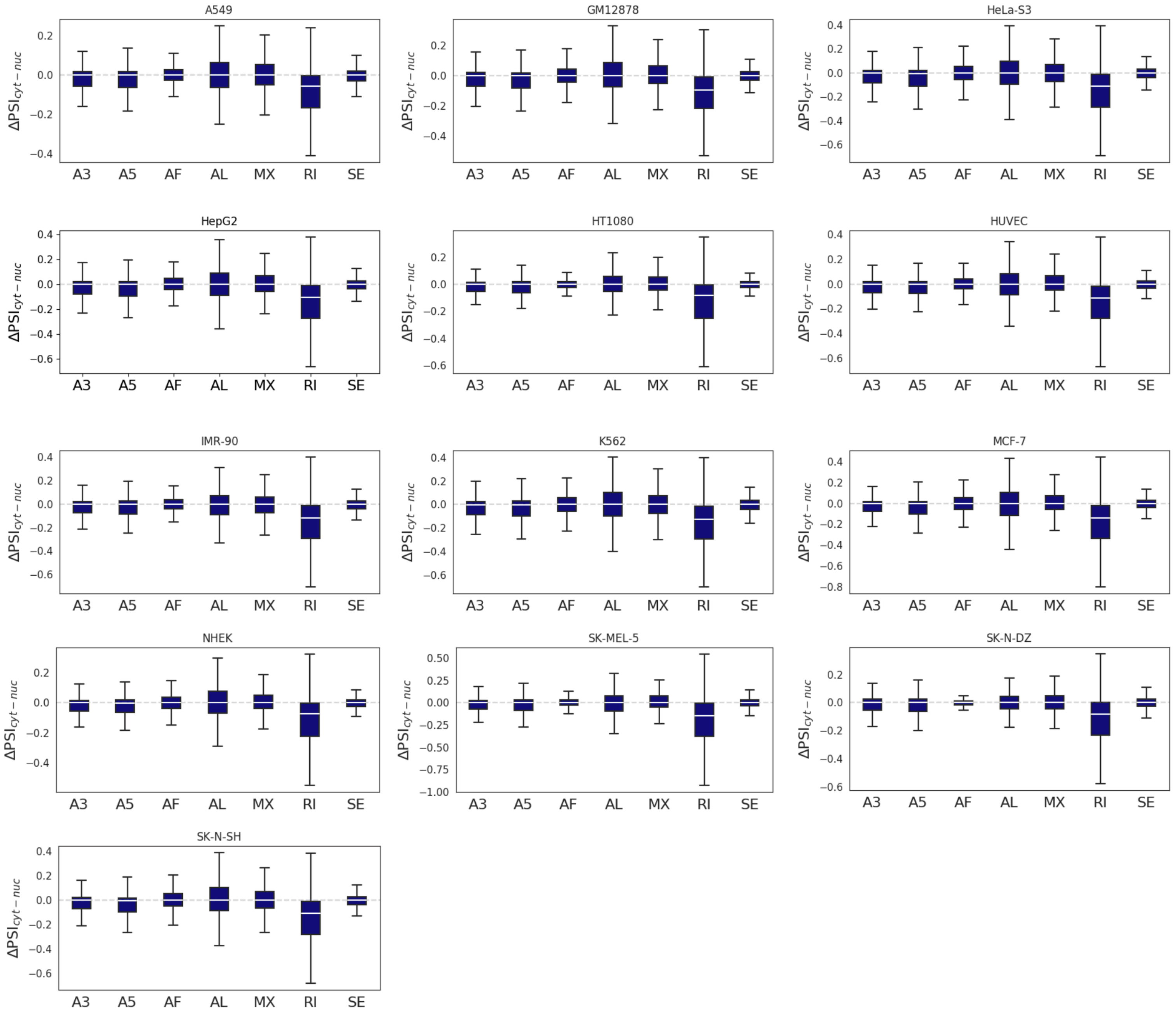

Supplementary Data 9. Comparison of alternative splicing patterns between cytoplasmic and nuclear transcripts. (protein-coding genes, continued)

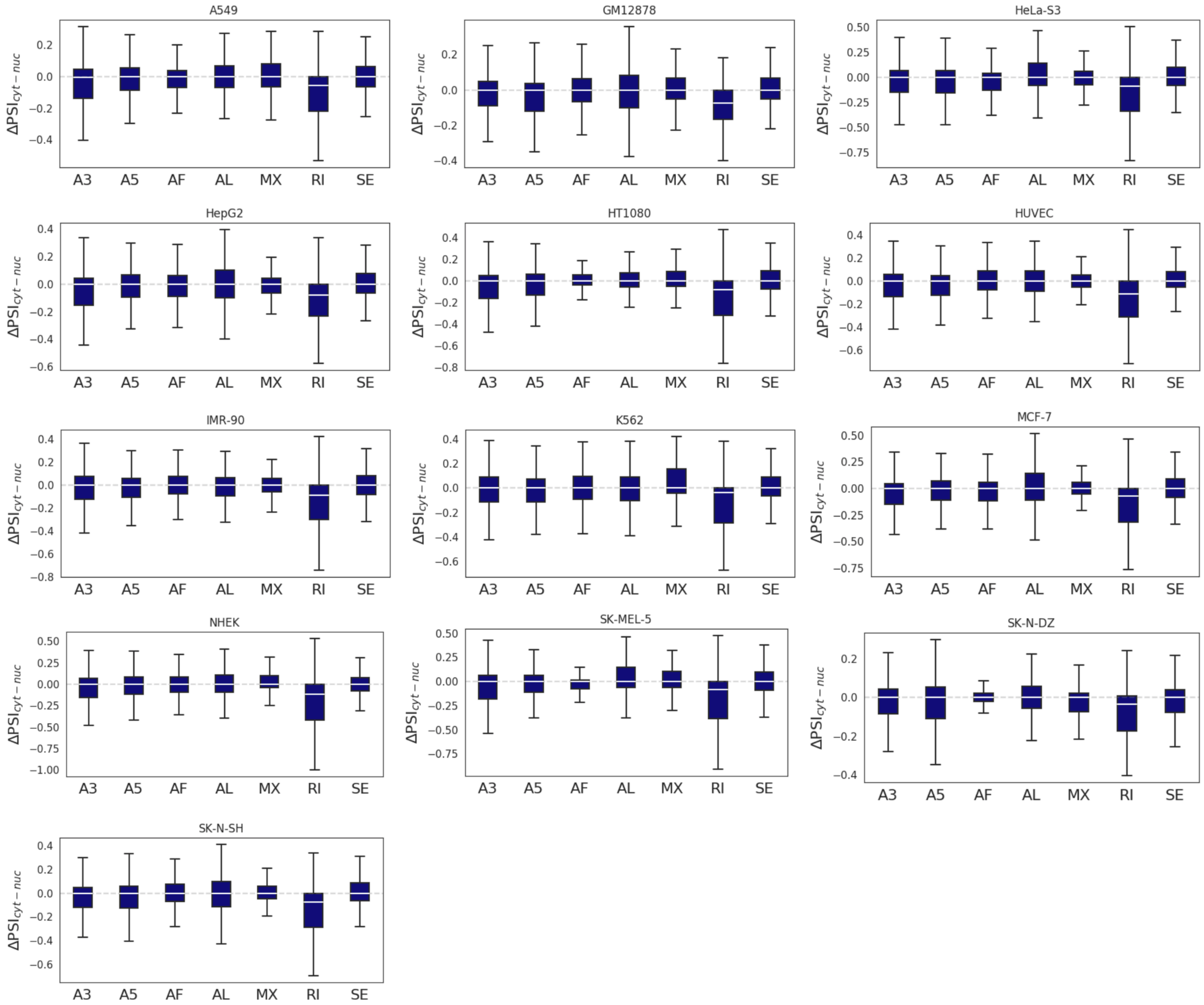

Supplementary Data 9. Comparison of alternative splicing patterns between cytoplasmic and nuclear transcripts. (noncoding genes)

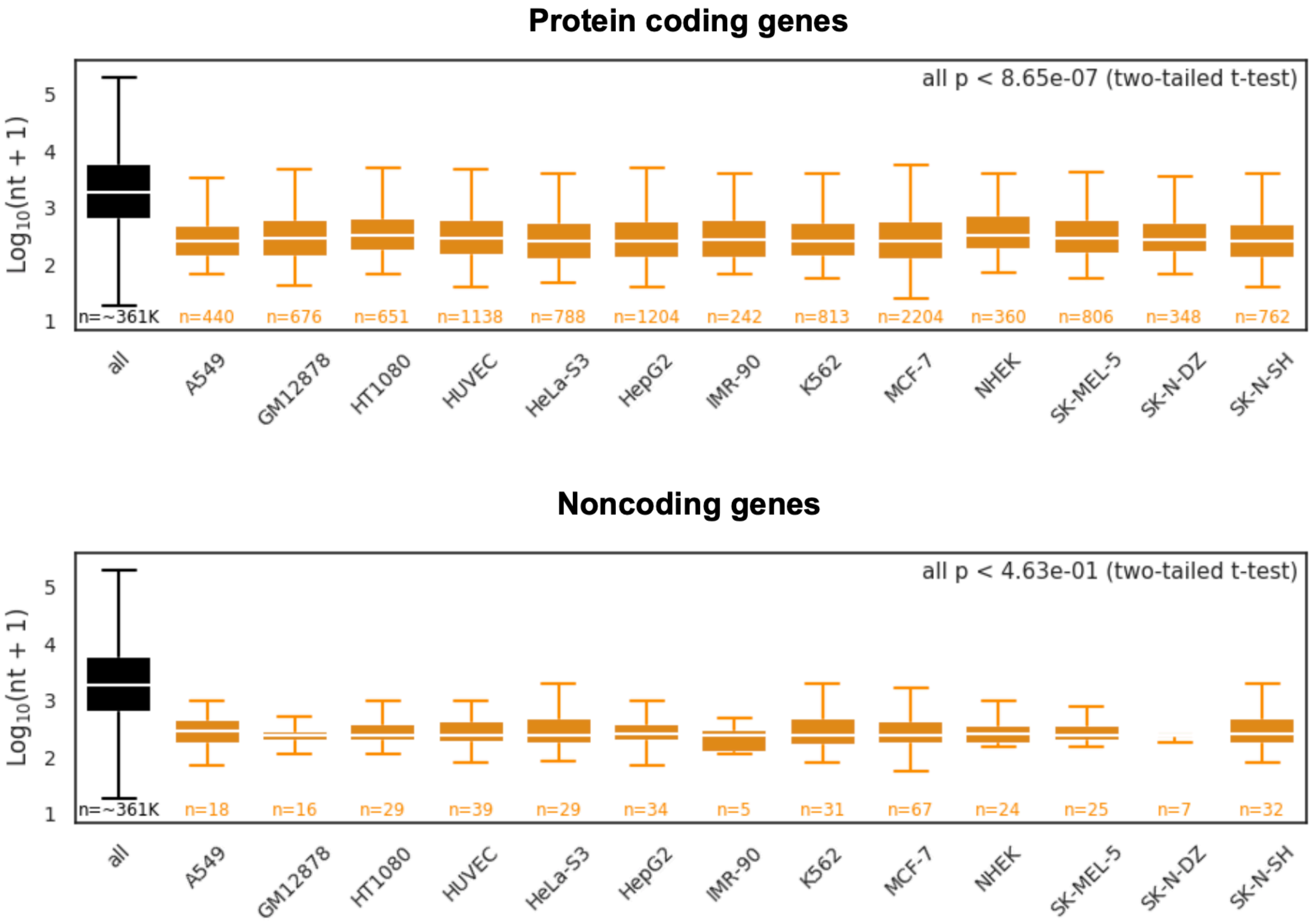

Supplementary Data 10. Comparison of length between all introns and nuclear RIs ($\Delta\Psi<0$ and $p<0.05$). Top and bottom panels for protein-coding and noncoding genes, respectively. P values: two-tailed t-test between all introns and nuclear RIs. The largest p value is 8.65e-07 and 4.63e-01 for protein-coding and non-coding genes, respectively.

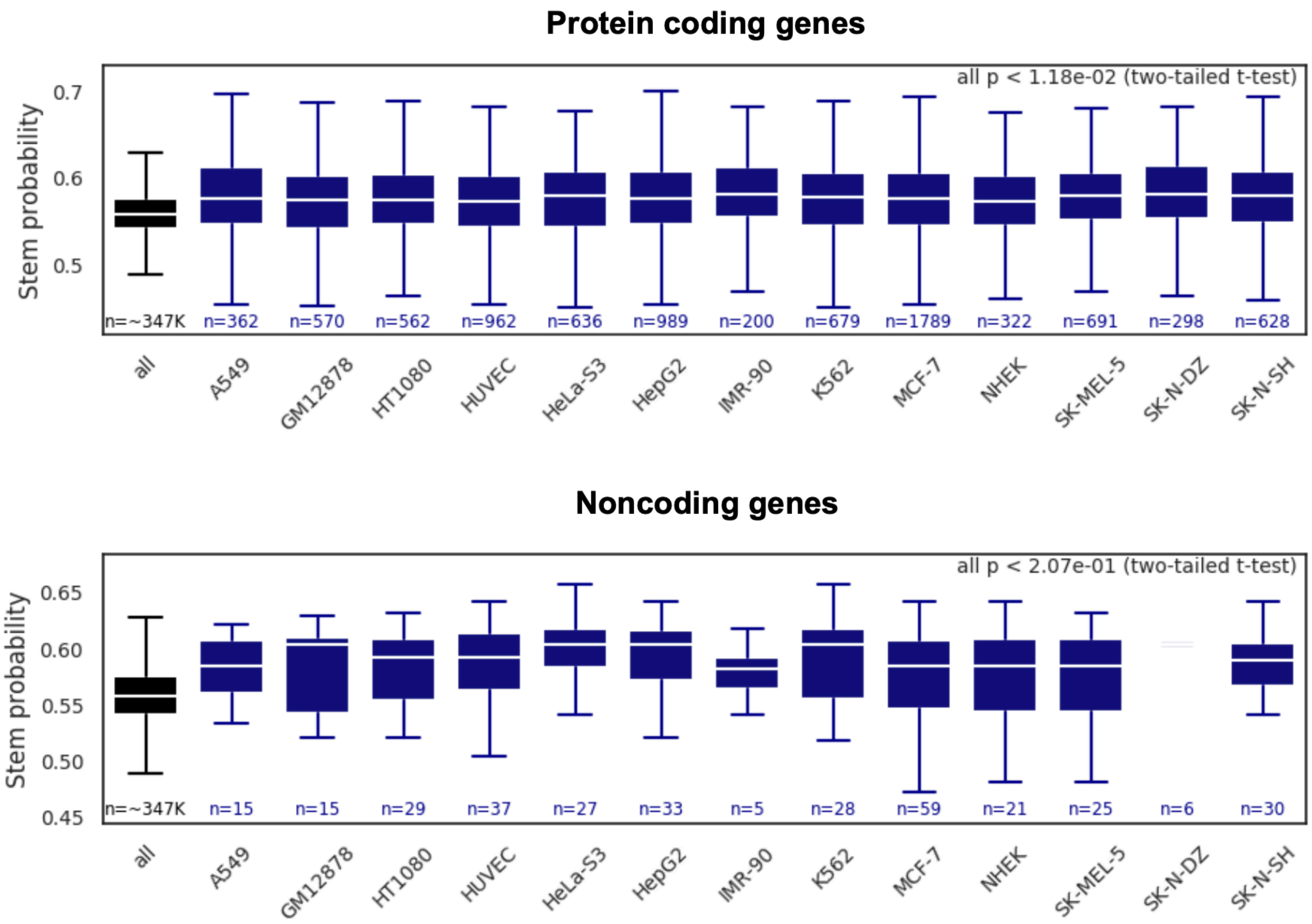

Supplementary Data 11. Comparison of RNA secondary structure (stem probability) between all introns and nuclear RIs (ΔΨ<0 and p<0.05). Top and bottom panels for protein-coding and noncoding genes, respectively. P values: two-tailed t-test between all introns and nuclear RIs. The largest p value is 1.18e-02 and 2.07e-01 for protein-coding and non-coding genes, respectively.
